# Up-regulation of CNDP2 facilitates the proliferation of colon cancer

**DOI:** 10.1186/1471-230X-14-96

**Published:** 2014-05-21

**Authors:** Conglong Xue, Zhenwei Zhang, Honglan Yu, Miao Yu, Kaitao Yuan, Ting Yang, Mingyong Miao, Hanping Shi

**Affiliations:** 1 Department of Surgery, The First Affiliated Hospital, Sun Yat-sen University, 58 Zhongshan II Road, Guangzhou, Guangdong 510080, China; 2 Department of Biochemistry and Molecular Biology, Second Military Medical University, 800 Xiangyin Road, Shanghai 200433, China; 3 Department of Intensive Care Unit, Zhongshan people’s Hospital, 2 Sunwen East Road, Zhongshan, Guangdong 528403, China

**Keywords:** CNDP2, Colon cancer, Poliferation, Clinicopathological Characteristics, RNA interference

## Abstract

**Background:**

Cytosolic nonspecific dipetidase (CN2) belongs to the family of M20 metallopeptidases. It was stated in previous articles that higher expression levels of CN2 were observed in renal cell carcinoma and breast cancer. Our study explored the correlation between CN2 and colon carcinogenesis.

**Methods:**

We analysed the relationship between 183 patients clinicopathological characteristics and its CN2 expression. To detect the levels of CN2 in colon cancer cell lines and colon cancer tissues by western blot. To verify cell proliferation in colon cancer cells with knockdown of CNDP2 and explore the causes of these phenomena.

**Results:**

The expression levels of CN2 in clinical colon tumors and colon cancer cell lines were significantly higher than that in normal colon mucosa and colon cell lines. The difference in CN2 levels was associated with tumor location (right- and left-sided colon cancer), but there was no significant association with age, gender, tumor size, tumor grade, tumor stage or serum carcinoembryonic antigen (CEA). Knockdown of CNDP2 inhibited cell proliferation, blocked cell cycle progression and retarded carcinogenesis in an animal model. The signaling pathway through which knockdown of CNDP2 inhibited cell proliferation and tumorigenesis involved in EGFR, cyclin B1 and cyclin E.

**Conclusions:**

Knockdown of CNDP2 can inhibit the proliferation of colon cancer in vitro and retarded carcinogenesis in vivo.

## Background

Colorectal cancer (CRC) is the third most commonly diagnosed cancer in males and the second most common in females, with over 1.2 million new cancer cases and 608,700 deaths estimated to have occurred in 2008 [1]. CRC was categorized into either proximal (right) or distal (left) location relative to the splenic flexure. This method appears very simple, but cumulative evidence found that underlying molecular features are different in right- and left-sided colon cancers. Bufill *et al.* was the first to comprehensively propose that colon cancer found in the proximal and distal locations of the colon may follow different biological pathways [2]. The reason for this difference is uncertain, but it could partly be explained by the different embryological development of the two segments of the colon, which may result in different genetic mutants or other molecular biological patterns of tumors, which therefore represent two separate disease entities [3].

Cytosolic nonspecific dipeptidase (CN2), named tissue carnosinase previously, belongs to the family of M20 metallopeptidases. It degrades carnosine (β-alanyl-L-histidine), which is an important bioactive dipeptide [4]. However, there are contradictory arguments on the function of CN2 in degrading carnosine. The study of Teufel *et al.* showed that little activity of CN2 was detectable under physiological conditions [4], whereas Pandya *et al*. suggested that carnosine can be hydrolyzed by CN2 under physiological conditions [5]. An HPLC-based method was used in the former study to detect the activity of CN2 while an MS-based assay in the latter study. The different methods might lead to the contradictory conclusions. Many researches showed that carnosine can inhibited the growth of malignant cells. Carnosine was recently reported that it retarded tumor growth *in vivo* mouse models and inhibited the proliferation of colon cancer cell *in vitro*[6,7]. The activity of CN2 can strongly be inhibited by bestatin [4]. A randomized phase III study of bestatin as a postoperative adjuvant treatment in patients with stage I squamous cell lung cancer was performed, and statistically significant clinical improvement in overall survival and disease-free survival was ascertained [8]. However, the mechanisms of the antitumor effects of bestatin and carnosine are not fully understood. Current studies about the relationship between CN2 and cancer have not come to an accordant conclusion. The expression levels of CN2 were high in breast cancer and kidney cancer but low in pancreatic cancer and hepatocellular carcinoma [9-13]. Thus, the research on the correlation between CN2 and colon cancer could reveal the mechanism driving colon cancer growth.

In this study, we examined CN2 expression in colon mucosa (CM), benign colon diseases (BCD) and colon cancers (CC). We also examined the effect of CN2 in tumor cell growth, colony formation and colon cancer cell tumorigenicity in nude mice.

## Methods

### Patients and specimens

One hundred fifty colon cancer samples and corresponding normal colon samples (at least 5 cm away from the tumor margin) and thirty three colon polyp were obtained and pathologically confirmed at the first affiliated hospital of Sun Yat-Sen University. 150 samples came from the patients with colon adenocarcinom who underwent curative resection between 2007 and 2009, and the other 33 ones were obtained from the patients with benigh colon diseases (BDC) by colonoscopy. The clinical characteristics of all these patients are summarized in Table 1. Clinical stages were classified according to the TNM stages of colorectal cancer defined by the Union for International Cancer Control (UICC). Eight tumor tissues and corresponding normal colon mucosa tissues were used for western blotting analysis. All samples were anonymously coded in accordance with local ethical guidelines, and written informed consent was obtained. This study was approved by the Review Board of Sun Yat-Sen University.

**Table 1:** Association between clinicopathological variables and CNDP2 expression in 150 patients with CC

**Variables**	**n (%)**	**CNDP2 expression**		***P *** **value**
		**Low**	**High**	
Age — yr	Mean 57.5	56.2 ± 10.7	58.5 ± 9.5	0.894^a^
Gender—n (%)				
Male	76 (50.7)	29 (44.6)	47 (55.3)	0.195^b^
Female	74 (49.3)	36 (55.4)	38 (44.7)	
Tumour size —n (%)				
≤ 5 cm	44 (29.3)	22 (33.3)	22 (26.2)	0.340^b^
> 5 cm	106 (70.7)	44 (66.7)	62 (73.8)	
Lymph nodes metastasis—n (%)				
No	76 (50.7)	38 (57.6)	38 (45.2)	0.134^b^
Yes	74 (49.3)	28 (42.4)	46 (54.8)	
Tumour grade—n (%)				
1	20 (21.5)	8 (32.4)	12 (7.1)	0.434^b^
2	106 (53.9)	50 (46.0)	56 (64.3)	
3	24 (24.6)	8 (21.6)	16 (28.6)	
Tumor stage—n (%)				
I	24 (16.0)	12 (18.2)	12 (14.2)	0.927^b^
II	46 (30.7)	20 (30.3)	26 (31.0)	
III	62 (41.3)	26 (39.4)	36 (42.9)	
IV	18 (12.0)	8 (12.1)	10 (11.9)	
CEA—n (%)				
Negative	96 (64.0)	44 (63.8)	52 (64.2)	0.956^b^
Positive	54 (36.0)	25 (36.2)	29 (35.8)	
Tumor location—n (%)				
RCC	84 (56.0)	28 (42.4)	56 (66.7)	0.003^b*^
LCC	66 (44.0)	38 (57.6)	28 (33.3)	
RCC—n (%)				
Male	42 (50.0)	9 (33.3)	33 (57.9)	0.035^b*^
Female	42 (50.0)	18 (66.7)	24 (42.1)	
LCC—n (%)				
Male	34 (51.5)	20 (52.6)	14 (50.0)	0.833^b^
Female	32 (48.5)	18 (47.4)	14 (50.0)	

^a^Mann-whitney u test.

^b^Pearson Chi-Square test.

*Statistically significant.

### Immunohistochemistry

Paraffin sections (4 mm thick) were deparaffinized and rehydrated followed by immersing in 3% H_2_O_2_ to quench endogenous peroxidase activity. Then, the sections were treated with 0.01 M citrate buffer (pH 6.0) and blocked with 5% normal goat serum, followed by incubation with a monoclonal anti-CNDP2 antibody (1:400; Proteintech, Chicago, IL) overnight at 4°C. These primary antibodies were diluted in PBS buffer containing 5% normal goat serum. The negative control for each slide was incubated with 5% normal goat serum without primary antibody. The sections were then incubated with HRP-conjugated anti-rabbit IgG for 60 min at room temperature and imaged with a ChemMate TM Envision TM Dectection Kit (DAKO). Scoring was performed by two pathologists.

### Cell culture

The following human colon normal and cancer cell lines were used: FHC was purchased from the American Type Culture Collection; DLD-1, LS174T, LoVo, RKO HCT116, HT29 and SW480 were provided by the Institute of Biochemistry and Cell Biology of the Chinese Academy of Science. All cells were maintained in RPMI 1640 (Biowest) medium supplemented with 10% FBS (Biowest), 100 U/ml penicillin, 100 μg/ml streptomycin sulphate, and 1 mM sodium pyruvate at 37°C in 5% CO_2_. The cells were trypsinized with trypsin-EDTA.

### Knockdown of CNDP2 by RNA interference (RNAi)

Three RNAi candidate target sequences to human CNDP2 (Additional file 1: Table S1) were synthesized according to the structure of a GV112 (hU6-MCS-CMV-Puromycin) viral vector (Genechemgene, Shanghai, China) and then inserted into a linearized vector. RKO cells were subcultured at 1 × 10^5^ cells per well in 6-well tissue culture plates. After 24 h culture, cells were infected by recombinant lentivirus vectors at a multiplicity of infection (MOI) of 10 with Enhanced infection solution and cultured in RPMI-1640 medium containing 10% FBS. These cells were then selected with puromycin (Sigma-Aldrich, Cat. P9620) for 48 h. Puromycin-resistant clones were generated and screened using western blot analysis.

### Western blot analysis

Proteins from cells or fresh tissues were extracted in RIPA lysis buffer containing a protease inhibitor cocktail (Roche). The supernatant was collected, and the protein concentration was quantified using a protein assay reagent (BCA, Beyotime, Shanghai). The proteins were separated by SDS-PAGE, transferred to a nitrocellulose membrane and incubated with the monoclonal antibody anti-CNDP2 (Proteintech, Chicago, IL), Cyclin E, Cyclin B1, EGFR, phospho-ERK1/2, ERK1/2, phospho-AKT, AKT, phospho-mTOR and mTOR (Cell Signaling Technology, Beverly, USA) at 4°C overnight. Thereafter, membranes were incubated with HRP-conjugated anti-rabbit IgG (1:5000) for 60 min at room temperature. The reactions were visualized using enhanced chemiluminescence and detected on photographic film.

### Cell growth assay

The cells were seeded in a 24-well plate at a density of 8000 cells/well and then cultured for 6 d. Cells were trypsinized and counted every 24 h.

### Colony formation

The cells (3 × 10^2^) were plated onto a 6-well plate. Plated cells were incubated at 37°C for 10 d. The plates were then stained with crystal violet, and colonies containing more than 50 cells were counted.

### Flow cytometry

Ethanol-fixed cells were incubated with RNase A (100 mg/ml) for 30 min at 37°C and propidium iodide (PI, 50 mg/ml) for 30 min at 4°C. PI fluorescence was measured in a FACScalibur flow cytometer (BD). Data were collected from 10,000 single-cell events, and cell cycle phase distributions were calculated using MODFIT software (Verity Software House).

### Tumorigenicity assay in nude mice

The tumor formation ability of CNDP2-transfected RKO cells was evaluated by injecting cell suspensions into BALB/c nude male mice. For each mouse, 1 × 10^6^ cells of RKO cell (shRNA_CNDP2 or vector-transfected) were injected into buttocks. After 4 to 5 weeks, mice were sacrificed, and each tumor was dissected for weighing. Each experimental group consisted of five mice, and the experiment was repeated twice. All animal experiments were performed with institutional approval and conformed to the Cancer Research guidelines for the welfare of animals in experimental neoplasia at Sun Yat-Sen University.

### Statistical analysis

Baseline characteristics of patients and the levels of CN2 in colon tissue were compared using Student’s T-test (continuous variables) and Chi-squared (χ^2^) tests (categorical variables). All statistical tests and corresponding P-values reported were for two-sided tests, and statistical analysis was performed using SPSS software version 12.0. P-values < 0.05 were considered statistically significant.

## Results

### CN2 expression in colon cancers and cells

**Figure 1:**
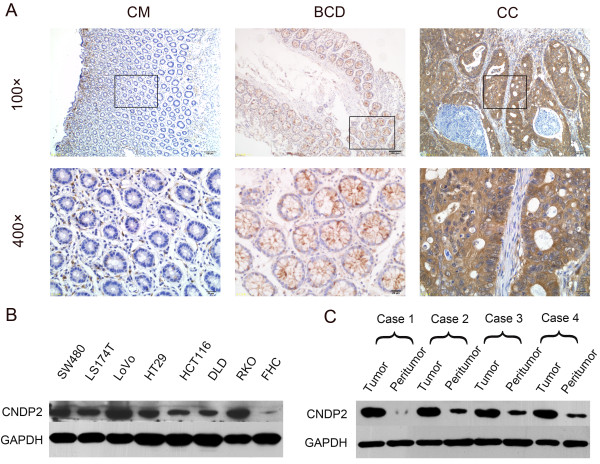
**CN2 expression in clinical colon tumors and cell lines. (A)** CN2 expression in CM, BCD and CC tissues.. Scale bars = 20 μm. **(B)** Western blot of CN2 in colon cancer cells and normal cells. **(C)** Western blot of CN2 in CC tissues and peritumoral tissues.

To explore the function of CN2 in colon cancer, we investigated CN2 expression in colon cancer tissues and peritumoral tissues. One hundred eighty-three samples from patients with colon disease, including 33 BCD samples, 150 CC samples and 150 CM samples were examined by immunohistochemistry. The CM samples consisted of peritumoral tissues that were resected at least 5 cm away from the tumor margin. The peritumoral epithelium and BCD displayed very weak staining and CC displayed very strong staining (Figure 1A) in the stained samples, and these samples displayed mainly CN2 expression in the cytoplasm (Figure 1A). 89.7% (126 out of 150) of the CM showed weak staining, while 75.8% (25 out of 33) of BCD expressed CN2 at low levels; 24.2% (8 out of 33) had higher levels. Notably, 50.7% (76 out of 150) of the CC samples displayed very strong cytoplasmic staining (Table 2 and Figure 1A). CN2 expression was significantly higher in CC than in BCD (p < 0.006) or CM tissues (p < 0.0001). In BCD tissues, CN2 expression was slightly higher than in CM; however, there was no statistical differences between the two (p = 0.259). In contrast, CM displayed very weak or negative staining (Figure 1A). Thus, CN2 immunostaining in CC was stronger than in their peritumoral counterparts and BCD. CN2 expression is markedly up-regulated in colon cancer. To further confirm our hypothesis, we examined CN2 expression in colon epithelial cells. Eight types of colon cells, including seven colon cancer cell lines (RKO, HCT116, DLD-1, LS174T, HT29, SW480 and LoVo) and the normal colon epithelial cell line (FHC), were collected. CN2 expression was extremely low in FHC cells, while all seven colon cancer cell lines displayed high CN2 expression (Figure 1B). CN2 expression in CC tissues and their peritumoral counterparts was also compared by western blotting. CN2 expression was significantly higher in tumors than in their peritumoral counterparts (Figure 1C).

**Table 2:** Association between CNDP2 expression and groups of colon disease

**Groups**		**CNDP2 expression**		***P *** **value**
	**n (%)**	**Low**	**High**	
CM	150 (100)	126 (89.7)	24 (10.3)	<0.0001^a*^
BCD	33 (100)	25 (75.8)	8 (24.2)	
CC	150 (100)	74 (49.3)	76 (50.7)	
CM vs.BCD				0.259^b^
BCD vs. CC				0.006^b*^
CM vs. CC				< 0.0001^b*^

^a^Cochran-Mantel-Haenszel test.

^b^Pearson Chi-Square test.

*Statistically significant.

### Association of CN2 expression with clinicopathological parameters

The clinical characteristics of 150 patients with CC and the correlation of baseline characteristics with CN2 expression were reviewed. CN2 expression was significantly associated with tumor location, but there was no significant association with age, gender, tumor size, tumor grade, tumor stage or serum CEA by statistical analysis (Table 1). There were 84 right-sided colon cancers (RCC) and 66 left-sided colon cancers (LCC) in all 150 patients. CN2 expression in RCC was significantly higher than in LCC (p = 0.003, Table 1). CN2 expression in male RCC was stronger than in female RCC (p = 0.035, Table 1), but there was no statistical difference between male LCC and female LCC (p = 0.833, Table 1).

### CN2 is associated with cell proliferation, cell cycle progression and tumorigenicity

**Figure 2:**
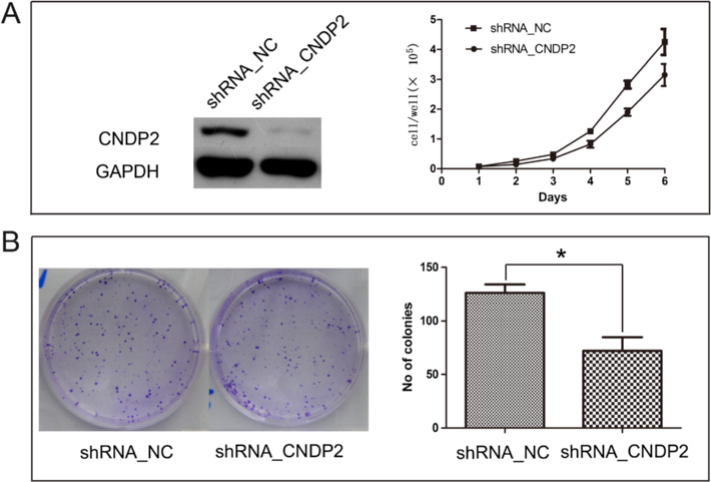
**Association of CN2 expression with colon cancer cell proliferation and colony formation. (A)**. RKO cells transfected with shRNA_CNDP2 showed a reduced growth rate. **(B)** RKO cells transfected with shRNA_CNDP2 showed reduced colony formation as compared to the controls. *p < 0.05.

**Figure 3:**
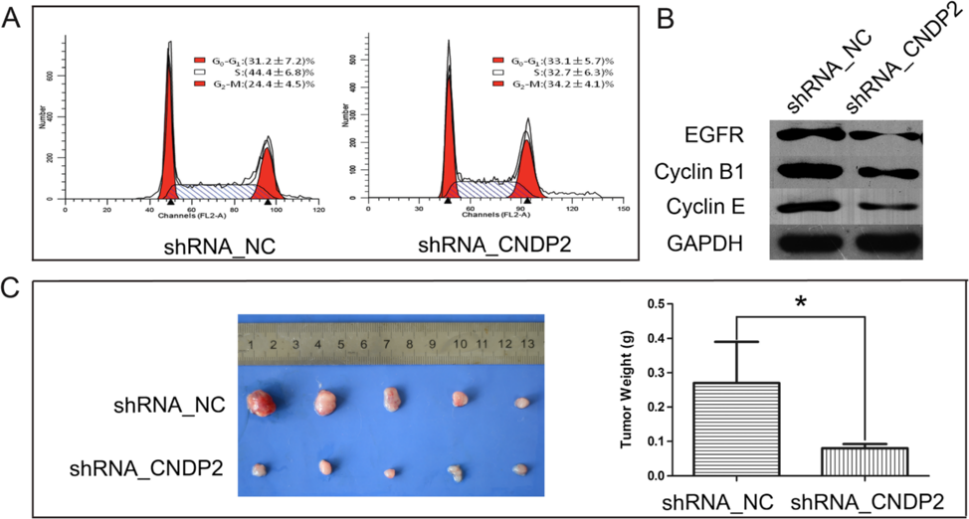
**Association of CN2 expression with colon cancer cell cycle progression and tumorigenicity. (A)** Cell-cycle phase distributions were analyzed by a FACScalibur flow cytometer. These experiments were repeated 3 times, and the symbols represent the mean values of triplicate tests (mean ± SD). **(B)** Western blotting analysis of cyclin E, cyclin B1 and EGFR expression. The experiment was independently repeated at least 3 times. **(C)** Knockdown of CNDP2 inhibited tumor formation in nude mice. Nude mice were inoculated with RKO stably transfected with empty vector and shRNA_CNDP2 expression vectors. *p < 0.05.

**Figure 4:**
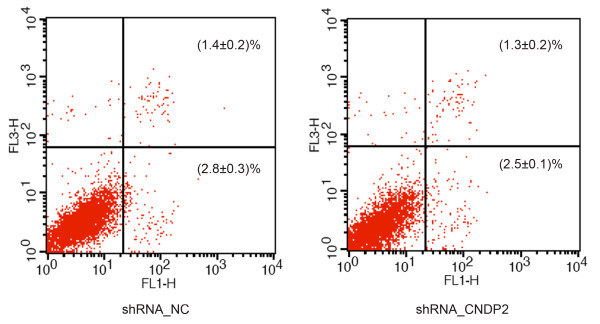
**Association of CN2 expression with cell apoptosis.** Cell apoptosis was analyzed by a FACScalibur flow cytometer. These experiments were repeated 3 times, and the symbols represent the mean values of triplicate tests (mean ± SD).

**Figure 5:**
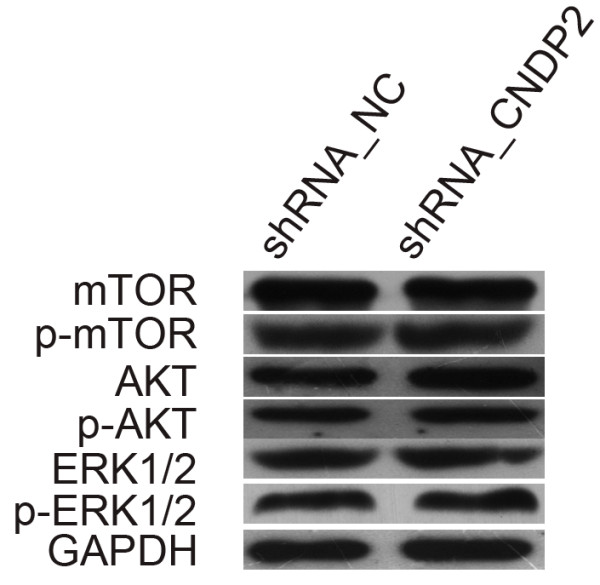
**Western blotting analysis of phospho-ERK1/2, ERK1/2, phospho-AKT, AKT, phospho-mTOR and mTOR expression.** The experiment was independently repeated at least 3 times.

In order to analyze the effects of CN2 on proliferation, we knocked down endogenous CNDP2 in RKO with shRNA_CNDP2. Knockdown of CNDP2 significantly inhibited the proliferation of RKO (p < 0.01, Figure 2A). After knockdown of CNDP2, the colony number was decreased (p < 0.01, Figure 2B). Moreover, RKO transfected with shRNA_CNDP2 had a larger fraction of the population in the G2/M phase (p < 0.05, Figure 3A) and a reduction in the proportion in S phase (p < 0.05, Figure 3A). Knockdown of CNDP2 reduced the S-phase fraction (SPF) and the number of cells arrested in G2/M. However, knockdown of CNDP2 did not affect apoptosis (Figure 4). Thus, knockdown of CNDP2 inhibited the proliferation of colon cancer cells. Additionally, knockdown of CNDP2 resulted in the decrease of cyclin E, cyclin B1 and EGFR expression in RKO cells (p < 0.05, Figure 3B), while there were no statistical differences in phospho-ERK1/2, ERK1/2, phospho-AKT, AKT, phospho-mTOR and mTOR expression (Figure 5). Furthermore, knockdown of CNDP2 retarded RKO tumorigenicity in nude mice (p < 0.05, Figure 3C).

## Discussion

CN2 expression exists in different human tissues ubiquitously [4]. The pathophysiological relevance of CN2 is degradation of carnosine (β-alanyl-L-histidine), which is an important bioactive dipeptide. However, there are contradictory arguments about the function of CN2. Teufel *et al*. stated that CN2 showed little bioactivity at pH7.5 and degraded carnosine at pH9.5, whereas Pandya *et al*. suggested that CN2 could degrade carnosine under physiological conditions and there was no significant change in the activity of CN2 with the change of pH [5]. An HPLC-based method was used in the former study to detect the activity of CN2 while an MS-based assay in the latter study. The different methods might lead to the contradictory conclusions. Biochemically, carnosine has the properties of pH-buffering, metal-ion chelation, antioxidant, and the capacity to defend against formation of advance dglycation and lipoxidation end-products. These properties determine its physiological roles [14].

In our study, we found that CN2 expression is dramatically increased in clinical colon cancer samples and colon cancer cells. In clinical samples, CN2 expression was significantly higher in CC than in BCD and CM tissues. In colon cell lines, CN2 expression was statistically higher in RKO cells as compared to normal FHC colon cells. These results suggest that high CN2 expression is associated with tumorigenesis. Furthermore, we found that CN2 expression in RCC is remarkably higher in comparison to LCC.

CC was divided into RCC and LCC relative to the splenic flexure. RCC consists of the cecum, ascending colon, hepatic flexure, and transverse colon. LCC consists of the splenic flexure, descending colon and sigmoid colon. There were many differences in pathological characteristics, clinical symptoms and biological features. Cancers on the right side tend to be exophytic and present with symptoms such as anemia, and left-sided cancer tends to be circumferential and can obstruct the bowel. Several groups have continued to develop the theme of differences between right- and left-sided colon cancers, and they have suggested that there are differences in epidemiology, clinical manifestation, pathology and prognosis between patients with right- and left-sided colon cancer [2,15]. RCC is more likely to be detected at an advanced stage with severe symptoms, and survival is significantly worse in patients with right-sided cancer [16,17]. However, the impact of tumor location itself on status and survival remains uncertain. Our results showed that CN2 expression in RCC was significantly higher than in LCC, which could explain why there are many different biological characteristics in the two segments of colon cancer. Moreover, we also found CN2 expression in male RCC was stronger than in female RCC, but there was no statistical difference between male LCC and female LCC. This result may be due to a small sample number.

Tripathi *et al.* reported that CN2 expression was remarkably higher in breast carcinomas *in situ* than normal breast cancer tissues and reduced mammoplasty breast tissues [9]. Two other studies demonstrated high expression of CN2 in kidney cancer tissues [10,11]. Our results agree with these studies, and several lines of evidence suggest that down-regulation of CNDP2 inhibits the proliferation of colon cancer. First, the expression levels of CN2 was significantly associated with EGFR expression, and EGFR serves as a marker of cell proliferation, migration and metastasis. Thus, elevated CN2 expression is likely associated with the proliferation of colon cancer cell. Second, down-regulation of CNDP2 in colon cancer cells inhibited cell proliferation and influenced cell cycle. Finally, down-regulation of CNDP2 inhibited tumor formation in nude mice. Collectively, these data suggest a role for CN2 in cell proliferation, but there are opposing views about CN2. Lee *et al.* reported that the CNDP2 gene and its splicing variant CPGL-B serve as growth-suppressor genes in pancreatic cancer [12]. Zhang *et al.* reported that CPGL-B significantly inhibits cell viability, colony formation, cell invasion and tumor formation in nude mice [13]. These contradictory results could be explained by differences in organization and cell type.

The signaling pathway through which CN2 promotes cell proliferation and tumorigenesis is unknown. We found that cyclin E expression was significantly lower after knockdown of CNDP2, which resulted in the reduction of SPF. Cyclin E, the essential S-phase kinase in Drosophila, promotes the G1/S-phase transition. Cyclin E shows cyclic expression and accumulates only during late G1, where it associates with CDK2 and promotes entry into S phase [18,19]. Amplification of cyclin E and CDK2 genes play a role in colorectal tumorigenicity [20]. To estimate the rate of cell proliferation, the SPF was frequently used in breast cancer, thyroid cancer and oral leukoplakia [21-23]. In colon cancer, high SPF was significantly associated with a poor prognosis [24,25]. We also found that knockdown of CNDP2 reduced cyclin B1 expression, which increased the levels of G2. Cells were arrested in G2/M. Cyclin B1 is one of the key regulators of the G2/M transition [26], and gene amplification or overexpression of cyclin B1 is associated with cell growth and tumorigenesis in colon cancer [27]. EGFR activation is required for progression from G2 to M phase [28]. Cyclin B1 expression is induced by various growth factors that could be activated via the EGFR/ERK pathway [29]. We found that suppression of CNDP2 blocked cell cycle progression and decreased the expression of cyclin E, cyclin B1 and EGFR in colon cancer cells. These results suggested that products of CN2 reaction may affect the expression of EGFR, cyclin B1 and cyclin E.

There are several limitations of our study. We could not determine the CN2 activity, which is crucial in pathophysiological process. The content of carnosine in the supernatant of proteins from fresh extracted tissues (colon cancer samples and corresponding normal colon samples) was measured by HPLC-based method and MS-based assay, however we got the negative results. So we don’t suggest that knockdown of CNDP2 inhibited cell proliferation is linked to the blunted degradation of carnosine. It is equally not clear whether there is the content of carnosine in colon cancer and colon mucosa. The further study will focus on the relationship between the activity of CN2 and colon cancer. We will also study whether CN2 expression will affect the prognosis of patients with colon cancer.

In conclusion, The strong CN2 expression that we observed in neoplastic colon cells and tumor tissues suggested a potential role for CN2 in colon tumorigenesis. Knockdown of CNDP2 retarded the growth of tumor cells and colony formation, and inhibited the tumorigenicity of colon cancer cell (RKO) in nude mice. Our work demonstrated that CNDP2 promoted the growth of cancer cells. We found that the expression of CN2 varied by colon location, which may provide a clue to the mechanism in right- and left-sided colon tumorigenicity. Our results suggested that CN2 could be a useful target for colon cancer therapy.

## Conclusions

Our results suggested that knockdown of CNDP2 inhibited cell proliferation *in vitro* and retarded carcinogenesis *in vivo*.

## Abbreviations

AKT: Serine/threonine-specific protein kinase; BCD: Benign colon diseases; CC: Colon cancers; CEA: Carcinoembryonic antigen; CDK2: Cyclin-dependent kinase 2; CM: Colon mucosa; EGFR: Epidermal growth factor receptor; ERK1/2: Extracellular-signal-regulated kinase 1/2; LCC: Left-sided colon cancer; mTOR: Mammalian target of rapamycin; RCC: Right-sided colon cancer.

## Competing interests

The authors have no competing interests to declare.

## Authors’ contributions

HPS, MY and CLX have conceived and designed the experiments, as well as have been involved in drafting the manuscript. CLX and ZWZ have performed the experiments. HLY and MY have participated inclinical data and information collection. CLX, KTY and TY have analyzed the date. All authors read and approved the final manuscript.

## Pre-publication history

The pre-publication history for this paper can be accessed here:

http://www.biomedcentral.com/1471-230X/14/96/prepub

## Supplementary Material

Additional file 1: Table S1 RNAi candidate target sequences for CNDP2. Click here for file
