# Supplementary material for: Up-regulation of CNDP2 facilitates the proliferation of colon cancer
Source: BMC Gastroenterol. 2014 May 21;14:96. doi: 10.1186/1471-230X-14-96 (PMC4035726; doi:10.1186/1471-230X-14-96)
Supplement: Additional file 1: Table S1 — RNAi candidate target sequences for CNDP2. [file 1471-230X-14-96-S1.doc]

Table S1. RNAi candidate target sequences for CNDP2.

| Sequence name | Sequence (5'-3') |
| --- | --- |
| NS-siRNA | TTCTCCGAACGTGTCACGT |
| CNDP2-8856 | CCGGCCTAACTAAGAAGTTTGCTGActcgagTCAGCAAACTTCTTAGTTAGGTTTTTG |
| CNDP2-8857 | CCGGCGACTTTGACATAGAGGAGTTctcgagAACTCCTCTATGTCAAAGTCGTTTTTG |
| CNDP2-8858 | CCGGGCAGCAACAAAGACCTCCATTctcgagAATGGAGGTCTTTGTTGCTGCTTTTTG |
